# Compliance of Interventional Radiologists With Interventional Oncology Accreditation Standards

**DOI:** 10.7759/cureus.42608

**Published:** 2023-07-28

**Authors:** Murat Dokdok, Huseyin Tugsan Ballı

**Affiliations:** 1 Radiology Department, Anadolu Medical Center, Kocaeli, TUR; 2 Radiology Department, Çukurova University School of Medicine, Adana, TUR

**Keywords:** interventialists, iasios, interventional radiology, interventional oncology, accreditation, standards of quality

## Abstract

Purpose: We aimed to evaluate interventional radiologists’ compliance with patient care and the quality management process of cancer patients using a national survey.

Methods: An electronic survey was designed with questions derived from the core criteria of the International Accreditation System for Interventional Oncology (IASIOS), with the approval of the IASIOS council. Among the interventional radiologists contacted by e-mail through the national association, 34 responded to the questionnaire. The agreement of the participants with the core requirements was evaluated in five questions consisting of 34 articles using the 5-point Likert scale.

Results: Regarding the years of experience in interventional radiology (IR), the mean scale for the less than five-year group was 118.4, while that for the group with more than 15 years was 145.17 (p = 0.030). The mean scale of the five- to 15-year-old group was 121.75, versus that of more than 15 years, which was 145.17 (p = 0.028). Thus, significant differences arose between <five years versus >15 years and five to 15 years versus >15 years groups; later groups were more likely to comply. There was also a statistical difference between the groups formed according to the ratio of oncological interventions (<25% vs. 25%-50%) in the daily workload (p = 0.010).

Conclusion: Increased experience in IR and more relay on oncological interventions appear to augment compliance with the IASIOS criteria. We believe that interventional radiologists who have distinct territorial praxis could benefit from such a framework with improved self-awareness.

## Introduction

Dealing with cancer diseases in interventional radiology (IR) requires a more specialized approach. Therefore, standardization of interventional oncology (IO), as a novel branch, might be needed all over the world. To deliver such services at a high level around the globe, standards of quality assurance have been proposed by the International Accreditation System for Interventional Oncology (IASIOS) [1]. We aimed to gain insight into the process of patient care and quality management in IO among interventional radiologists in Turkey, based on IASIOS criteria. Although interventional radiology has not been officially accepted as a subspecialty in Turkey, the Cardiovascular and Interventional Radiological Society of Europe (CIRSE)-endorsed diploma program and CIRSE curricula have been adopted by the Turkish Society of Interventional Radiology (TSIR) [2, 3]. As far as we know, this is the first survey that has investigated the compliance of interventionalist radiologists with IO services for accreditation.

## Materials and methods

An electronic survey was designed with questions derived from the core criteria of IASIOS accreditation, with the approval of the IASIOS council. After being translated into Turkish, the questionnaire was sent to the e-mails of member interventional radiologists with an announcement by TSIR. Preliminary information about maintaining the confidentiality of the participants was also included. Multiple responses were made impossible via the same Internet protocol (IP) address. The data of 34 interventional radiologists who participated were collected in an online survey database. Thus, the other professionals were excluded from this study. For this type of study, the formal consent of an ethics committee is not required at the authors’ institution.

Subgrouping of questions and interventionalists

The questions in the survey were categorized into two groups: the baseline characteristics of the participants and their compliance with the IASIOS criteria.

The first five questions regarding the baseline characteristics of participants included: the institution of employment; the certification or diploma in IR; the years of experience in IR; the ratio of IO to daily workload; and the number of procedures performed annually. The agreement of the participants with the IASIOS core requirements was asked in the last five questions, consisting of 34 articles, using the 5-point Likert Scale.

Although derived from all 28 core IASIOS criteria regarding staff and facilities, treatment planning and delivery, safety, and quality, the questions were not formed by point-by-point translation. For the ease of the survey, the personnel competence and workforce; the management of patient records and clinical data; the management of medical services and the processes related to the purchase of medical devices and consumables; the planning and delivery of IO services; and the quality and safety management of IO services were each assessed in five sections separately (Table 1).

**Table 1:** Questions derived from the core criteria of IASIOS accreditation [1]

Question groups
1. Regarding the personnel's (interventional radiologist, technician, nurse, etc.) competence and workforce in the institution where you work for IO (interventional oncology) services
a) Employees have the required diploma (national ones are compulsory for interventional radiologists), license, or certificates. b) Each employee regularly participates in continuous medical education (congresses, courses, and in-department training). c) A work schedule is kept in such a way as to ensure the continuity of work for employees who take annual leave and for the people who will work instead. d) Interventional oncology (IO) procedures are recorded properly; their nature and number can be determined annually. e) Before and after IO procedures, consultation, and follow-up notes are kept properly and on time (usually in the first 24 hours). f) Complications of IO procedures are recorded; morbidity and mortality meetings are held.
2. Regarding the creation and management of patient records and clinical data in your institution while providing IO services
a) We have written policies in line with international norms for keeping, archiving, and transferring patient records when necessary. b) Patient records are kept sufficiently clear (name, gender, date of birth, nationality, place of examination, and doctor) and up-to-date. c) The examination anamnesis forms of the patient include the reason for application, complaints if any, background (additional diseases and treatments), family history, nutritional status, and performance status. d) Current ICD disease codes (primary cancer) and cancer staging systems are applied to each patient. e) Cancer diagnosis date, diagnosis method, number of lesion sites, histological type, and differentiation were recorded. f) There are written procedures and instructions created for the treatment process of the patients. g) Patient information includes the procedure date, purpose (curative or palliative), technique and equipment used, and a written follow-up plan after the procedure, in accordance with the instructions.
3. Regarding the infrastructure of your institution for IO services, the management of medical services, and the management of processes related to the purchase of medical devices and consumables
a) For IO services, written policies are created for the implementation of a new process or technology in your institution. b) For IO services, management meetings (operational services, evaluation of performance indicators, risk, and safety records) are held and recorded at regular intervals in your institution. c) Periodic health and safety audits are made (internal and external), and the actions taken as a result are recorded. d) Multidisciplinary tumor meetings are always attended by an IO-interventional radiologist and recorded. e) An IO-interventional radiologist is always present and registered during the procurement process of new devices and materials for IO services. f) Acceptance tests of new devices and materials purchased for IO services are recorded. g) There is a written procedure for the purchase, storage, and management of medication, consumables, and equipment. h) There is a written procedure for detailed records and processes for reusable materials.
4. Regarding the planning and delivery of IO services in your institution
a) A consent form is obtained from all patients for each procedure, and possible changes that may occur during the procedure are indicated in the consent. b) There is a policy to verify the identity and a procedure that is documented. c) Before the interventions, a time-out or Cardiovascular and Interventional Society of Europe (CIRSE) checklist form is used. During and after the procedure, the patient's follow-up, monitoring, and vital signs are systematically evaluated and recorded. e) Systematic control of the equipment to be used before the operations is carried out and recorded in accordance with an existing written protocol. f) There is systematic control and records of all consumables and drugs before using them in the process.
5. Regarding the quality and safety management of IO services in your institution
a) A risk log has been recorded in which patient risks are analyzed and actions taken are indicated. b) There are records in which all kinds of incident notifications are made (including near misses) and actions taken accordingly are recorded. c) Feedback is provided to the employees regarding the incident notifications investigated, and these are recorded. d) Interventional radiologists who perform procedures involving ionized radiation doses and effects, in particular, have adequate training. e) There is a written policy on the approach to pregnant patients. f) All devices that are sources of radiation and radioactive material have appropriate registrations and licenses. g) The radiation safety training and dosimetry results of all personnel working in these areas are recorded.

The participants were not questioned on other criteria, such as scientific activities, as they were included in the extended criteria by IASIOS.

Statistical analysis

Descriptive statistics were presented as mean ± standard deviation, and minimum-maximum values for continuous variables and as numbers and percentages for categorical variables in the analysis of the scale. Cronbach's alpha coefficient was used for statistical validity and reliability analysis of the scale; a range between 0.81 and 1.00 was considered highly reliable. The assumption of normality was not met for the data after normality analysis. Therefore, the p-value of the Z test statistics was calculated using the Mann-Whitney U test, and the p-value of the Chi-Square test statistics was calculated using the Kruskal-Wallis test for the analyses of the scale. A p-value less than 0.05 was considered statistically significant. IBM Statistical Package for the Social Sciences (SPSS) Version 21.0 (Armonk, NY: IBM Corp.) was used for all calculations.

## Results

Baseline characteristics and background

Most of the participants were from university hospitals (35.29%), followed by those from private university hospitals (23.53%). They were mostly interventionalists for more than 10 years (55.88%), while only 29.41% of them were interventionalists for less than five years. While two-thirds (66.65%) of the participants had at least one certificate, most of the participants (61.76%) had been granted a certification from the Turkish Interventional Radiology Board. More than half of the respondents (55.88%) were performing oncological interventions in more than half of their daily workload. Biopsies were the most common procedures (mean 841 to 15,000), which were followed by fluid drainages (mean 403 and 0-2,000). The number of tumor treatments, such as ablation (mean 30.56 and range 0-250), chemoembolization (mean 37.38 and range 0-225), and radioembolization (mean 34.67 and range 0-750), varied among the participants. Pain procedures and the others were the least performed interventions.

Compatibility with the IASIOS criteria

Agreement with the IASIOS core criteria was assessed using the 5-point Likert Scale. To analyze the data, it is coded as follows: 1 = strongly disagree; 2 = disagree; 3 = neutral; 4 = agree; 5 = strongly agree. Within the five sections of the scale (mean scale ± standard deviation 129.03 ± 30,207), statistical validity and reliability analysis results using Cronbach's alpha coefficient demonstrated high reliability (0.974) overall.

Regarding the years of experience in IR, the mean scale for the less than five-year group was 118.4, while that for the group with more than 15 years was 145.17 (p = 0.030 <0.05). The mean scale of the five-15-year group was 121.75, versus that of more than 15 years, which was 145.17 (p = 0.028 <0.05). Thus, significant differences arose between the <five years versus >15 years and five-15 years versus >15 years groups; later groups were more likely to comply. The ratio of IO to daily workload revealed statistical significance between <25% and 25%-50% groups (p = 0.010 <0.05). However, for the agreement with IASIOS criteria, the number of procedures yielded no statistical significance, except for the biopsies.

After the data was divided into two nominal categories, such as agreed for 'strongly agree' and 'agree' responses and disagreed for 'neutral, 'disagree', and 'strongly disagree' responses, agreement on five groups of questions related to core criteria was analyzed. The highest agreement was on obtaining consent forms with an indication of possible changes during treatment (32 out of 34 participants, 94.12%), followed by that the devices that are sources of radiation and radioactive material have appropriate registrations and licenses (30 out of 34 participants, 88.53%). The lowest agreement was on the management meetings (operational services, evaluation of performance indicators, risk, and safety records) held and recorded at regular intervals in the institution (12 out of 34 patients, 35.29%), followed by agreement on a time-out or CIRSE checklist form used before interventions (14 out of 34 patients, 41.18%). However, the Chi-square test did not reveal any statistical difference (p<0.05) between or in the groups of five questions related to IASIOS core criteria (Table 2).

**Table 2:** Agreement with the IASIOS criteria based on different parameters SE: standard error; n: numbers; IR: interventional radiology; IO: interventional oncology

Parameters	Mean ± SE (min-max)	p-value
Institution		0,051
University (n = 12)	134,5 ± 8,0 (91-172)	
Private university (n = 7)	139,86 ± 10,61 (85-170)	
Educational hospital (n = 10)	104,4 ± 9,7 (75-163)	
Private hospital (n = 5)	144,0 ± 8,7 (124-169)	
Certification		0,164
No (n = 11)	118,82 ± 9,24 (75-165)	
Yes (n = 23)	133,91 ±6,13 (75-172)	
Years of experience in IR		0,041
<5 (n = 10)	118,4 ± 11,12 (75-165)	
5-15 (n = 12)	121,75 ± 7,46 (75-170)	
>15 (n = 12)	145,17 ± 9,4 (85-172)	
The ratio of IO in daily workload %		0,031
<50% (n = 7)	98,63 ± 9,385 (81-156)	
25-50% (n = 7)	144 ± 7,575 (115-167)	
51-75% (n = 10)	128,7 ± 10,745 (74-164)	
>75% (n = 9)	131,78 ± 4,910 (108-164)	
Biopsy (n) annually		0,021
<300 (n = 15)	140,87 ± 7,04 (85-172)	
300 and over (n = 19)	119,68 ± 6,82 (75-169)	
Drainage (n) annually		0,878
<100 (n = 16)	127,81 ± 8,11 (82-172)	
100 and over (n = 18)	130,11 ± 6,82 (75-170)	
Ablation (n) annually		0,126
<20 (n = 20)	121,11 ± 7,8 (75-170)	
20 and over (n = 14)	137,94 ± 5,83 (85-172)	
Embolization (n) annually		0,083
<20 (n = 20)	120,6 ± 7,55 (75-169)	
20 and over (n = 14)	141,07 ± 5,23 (111-172)	
Radioembolization (n) annually		0,058
<20 (n = 22)	121,18 ± 6,84 (75-170)	
20 and over (n = 12)	143,42 ± 5,91 (119-172)	

## Discussion

Accreditation is generally perceived as a part of an institutional process; however, it often does not address the specific needs of a particular service. A dedicated accreditation program might be useful, especially when dealing with a particular patient group or disease, such as cancer. The International Accreditation System for Interventional Oncology (IASIOS) is the first initiative to create a scheme that covers all aspects of organization in IO. We evaluated the compliance and degree of agreement of interventional radiologists in Turkey with the IASIOS core criteria in our study. The more years participants spent on the interventions, the more they showed agreement with the criteria. The ratio of the daily routine workload spent on IO was also correlating, as interventionalists who spent less than 25% were less likely to comply. The number of therapeutic procedures performed annually did not yield statistical significance for compliance among the interventionalists, whereas there is no such comparable data in the literature.

To ensure the quality and safety of patient care and maintain standardization and sustainable development, accreditation was introduced into healthcare services about two decades ago. Early studies demonstrated that hospital accreditation could induce professional development and promote change [4]. A positive impact of hospital accreditation was measured on safety culture, performance measures, efficiency, and patient length of stay in a recent large-scale systemic review [5]. On the other hand, the impact of specialty or disease-specific accreditation programs was also investigated in some studies [6-8]. Even tumor-based accreditation systems have been established in some countries [9]. Due to challenges in the provision of standard cancer care, accreditation programs were launched in Europe by the Organization of European Cancer Institutes and by the European Society of Medical Oncology in 2002 and 2003 [10,11]. Some credentialing organizations and regulatory requirements by government organizations already exist for diagnostic radiology; as such, the American College of Radiology has granted accreditation to over 40,000 radiology centers in the US [12]. As per the European Council Directive 2013/59/Euratom, a clinical audit is compulsory in the EU in accordance with national requirements. Based on the European Commission guidelines published for clinical audit, the European Society of Radiology Audit and Standards Subcommittee developed the ESR Clinical Audit Tool and published a booklet, Esperanto, to practice clinical audit in 2017 [13].

Although IO practice started in the 1970s, the first usage of IO terminology appeared in PubMed at the beginning of the 2000s. The Cardiovascular and Interventional Radiological Society of Europe (CIRSE), in the meantime, published the standards of practice guidelines for IO treatments and updated them occasionally [14]. The inclusion of IO in cancer treatment guidelines and recommendations is based on many research efforts in IO in recent years [15]. Some recommendations were also published regarding the provision of interventional radiology services in Europe [16]. However, they are essentially for interventional practice; they are neither comprehensive in all aspects nor impose any sanctions or an audit body.

Standards of quality assurance proposed by IASIOS could help to maintain the best medical practices in IO [3]. Currently, there are IASIOS-accredited centers in Europe, Asia, and Australia, and the numbers are increasing. According to a recent editorial manuscript from an IASIOS-accredited center, the framework of IASIOS may also offer benefits when expanded to the rest of the IR service [17]. We agree with the above conclusion of the authors, as there is still no national, regional, or internationally recognized accreditation system for IR. In this context, the second lowest compliance was on a time-out form in our study, although a similar CIRSE checklist was released more than a decade ago. A timeout form not being standard is not conceivable, and complying thoroughly may lead to better outcomes in terms of safety [18].

There are about 626 interventional radiologists who are registered as members of TSIR in Turkey. As a limitation, the sample for this study is less than 10%, which might not accurately reflect the actual data but could be informative. However, the database was still sufficient to withdraw meaningful statistical results. It could also be inferred that plenty of interventionalists who did not participate in the survey did not have any concerns or focus on oncological interventions when the number of member interventionalists was taken into account. A group that takes interventional oncology services seriously or vice versa might also be a limitation. Besides, other personnel who are a natural part of the accreditation process were not questioned in the survey. The participation of all healthcare professionals and ensuring their motivation are critically important to putting accreditation standards into practice [6]. Physicians were found to be more reluctant to participate in accreditation and to predict the benefits of accreditation [19]. As might be expected, the former participants believe strongly in the positive effect of accreditation on their hospitals in that study.

## Conclusions

In this study, we aimed to measure how prepared interventional radiologists are for patient care and the quality management process for cancer patients. This was accomplished through a national survey based on The Standards of Quality Assurance for Interventional Oncology, an initiative to establish the best medical practice for cancer patients in IO. It was ascertained that increased experience in IR and more focus on IO procedures appear to augment compliance with IASIOS criteria. Although interventional radiologists have distinct territorial praxis globally, such frameworks and improved self-awareness may help standardize interventions.
